# Ticagrelor as an Alternative Antiplatelet Therapy in Cardiac Patients Non-Sensitive to Aspirin

**DOI:** 10.3390/medicina56100519

**Published:** 2020-10-02

**Authors:** Hamzah Khan, Reid Gallant, Shubha Jain, Mohammed Al-Omran, Charles De Mestral, Elisa Greco, Mark Wheatcroft, Ashraf Alazonni, Rawand Abdin, Margaret L. Rand, Heyu Ni, Mohammad Qadura

**Affiliations:** 1 Division of Vascular Surgery, St. Michael’s Hospital, Toronto, ON M5B 1W8, Canada; hamzah.khan@mail.utoronto.ca (H.K.); jains@ucalgary.ca (S.J.); Mohammed.Al-Omran@unityhealth.to (M.A.-O.); CHARLES.DEMESTRAL@unityhealth.to (C.D.M.); Elisa.Greco@unityhealth.to (E.G.); Mark.Wheatcroft@unityhealth.to (M.W.); 2 Keenan Research Centre for Biomedical Science and Li Ka Shing Knowledge Institute of St. Michael’s Hospital, Toronto, ON M5B 1W8, Canada; reid.gallant@mail.utoronto.ca (R.G.); Heyu.Ni@unityhealth.to (H.N.); 3 Department of Surgery, University of Toronto, Toronto, ON M5T 1P5, Canada; 4 Division of Cardiology, Scarborough Health Network, Toronto, ON M1P 2T7, Canada; ashraf.alazzoni@medportal.ca; 5 Department of Medicine, McMaster University, Hamilton, ON L8N 3Z5, Canada; rawand.abdin@medportal.ca; 6 Department of Laboratory Medicine & Pathobiology, University of Toronto, Toronto, ON M5S 1A8, Canada; margaret.rand@sickkids.ca; 7 Departments of Biochemistry and Pediatrics, University of Toronto, Toronto, ON M5G 1X8, Canada; 8 Translational Medicine, Research Institute; Division of Haematology/Oncology, The Hospital for Sick Children, Toronto, ON M5G 1X8, Canada

**Keywords:** aspirin, light-transmission aggregometry, non-sensitivity, personalized medicine, atherosclerosis, antiplatelet therapy, resistance, ticagrelor

## Abstract

*Background and Objectives:* Aspirin (acetylsalicylic acid—ASA) is a first-line antiplatelet therapy provided to patients with coronary artery disease (CAD). However, it has been demonstrated that 20–30% of these patients are non-sensitive to their ASA therapy. ASA non-sensitivity is a phenomenon where low-dose ASA (81–325 mg) does not completely inhibit arachidonic-acid-induced platelet aggregation, putting patients at risk of adverse cardio-thrombotic events. Ticagrelor is a P2Y12 receptor inhibitor and alternative antiplatelet that has been approved to reduce the risk of stroke, myocardial infarction, and overall cardiovascular-related death. In this study, we aimed to identify ASA non-sensitive patients and evaluate if they would be sensitive to ticagrelor. *Materials and Methods:* For this pilot study, thirty-eight patients with CAD taking 81 mg ASA were recruited. Blood samples were collected from each patient and platelet rich plasma (PRP) from each sample was isolated. Light-transmission aggregometry (LTA) was used to determine baseline ASA sensitivity in each patient using 0.5 mg/mL arachidonic acid as a platelet agonist. Patients with ≥20% maximal platelet aggregation after activation were considered ASA non-sensitive. Fresh PRP samples from all patients were then spiked with a clinical dosage of ticagrelor (3 μM—approximately equivalent to a loading dose of 180 mg ticagrelor). Sensitivity was determined using LTA and 5 μM ADP as a platelet agonist. Patients with ≥46% maximal platelet aggregation were considered ticagrelor non-sensitive. *Results:* Of the 38 CAD patients taking 81 mg ASA, 32% (12/38) were non-sensitive to their 81 mg ASA therapy. All 38 of the recruited patients (100%) were sensitive to ticagrelor ex vivo. In conclusion, we were able to identify ASA non-sensitivity using LTA and determine that ASA non-sensitive patients were sensitive to ticagrelor. *Conclusions:* Our results suggest that ticagrelor is a promising alternative therapy for patients who are non-sensitive to ASA.

## 1. Introduction

Patients with atherosclerotic diseases such as coronary artery disease (CAD), are at a greater risk of adverse cardio-thrombotic events such as myocardial infarction, stroke, and ischemia [1,2,3,4,5]. Acetylsalicylic acid (ASA), better known by its commercial name aspirin, is a common medication used to treat fever, pain, and inflammation. In low doses, the antiplatelet effects of ASA have been well studied, and it is a cornerstone of secondary prevention of adverse cardio-thrombotic events in atherosclerotic patients [6,7,8,9]. The Antithrombotic Trialists Collaboration conducted a meta-analysis of 287 trials including almost 200,000 patients and demonstrated a 22 percent reduction in mortality and serious adverse cardio-thrombotic events in patients taking ASA [10]. However, research has demonstrated that between 20–30 percent of patients taking 81 mg ASA therapy are considered ASA non-sensitive, defined as the inability of low-dose ASA (81–325 mg) to completely inhibit arachidonic-acid-induced platelet aggregation [11,12,13,14,15,16,17]. A meta-analysis conducted by Krasopouls et al. in 2008 demonstrated that ASA non-sensitive patients had a significantly higher risk of death, acute coronary syndrome, failure of vascular interventions, or a new cerebrovascular event compared to sensitive patients [18].

Ticagrelor is an alternative antiplatelet therapy that is commonly prescribed to patients with acute coronary syndrome (ACS), defined as patients with unstable angina, non-ST-segment elevation myocardial infarctions (NSTEMI), or ST-segment elevation myocardial infarctions (STEMI), as well as patients with a previous history of myocardial infarction (MI) [19,20,21]. This medication binds to, and reversibly inhibits the P2Y12 adenosine diphosphate (ADP) receptor on platelets, inhibiting ADP-induced platelet aggregation [22]. The Effect of Ticagrelor on Health Outcomes in Diabetes Mellitus Patients Intervention (THEMIS) trial conducted in 2019 demonstrated that stable coronary artery disease patients and diabetics taking ticagrelor with ASA had a significant reduction in adverse cardio-thrombotic events when compared to patients on ASA and a placebo [23]. ASA in combination with ticagrelor simultaneously inhibits both the arachidonic acid and ADP platelet activation pathways [24,25]. Researchers, however, did note an increase in major bleeding in patients on dual antiplatelet therapy, demonstrating the need for sensitivity testing to ensure that stable CAD patients are receiving an antiplatelet they are responsive to, rather than dual antiplatelet therapy when not necessary [23].

Patients who are ASA non-sensitive are at a higher risk of further adverse cardio-thrombotic events, and may benefit from the prescription of an alternative antiplatelet therapy such as ticagrelor [18]. It is unclear whether ASA non-sensitivity alters the efficacy of other antiplatelet drugs. In this pilot study, we used light-transmission aggregometry to determine whether CAD patients who are non-sensitive to ASA would respond to ticagrelor.

## 2. Materials and Methods

### 2.1. Ethics Approval

This study was performed in accordance with the Declaration of Helsinki and approved by the Unity Health Toronto Research Ethics Board at St Michael’s Hospital, Toronto, Canada (REB #16–375, 8 February 2017). All patients provided written informed consent before participating in this study.

### 2.2. Patient Selection

Patients were recruited from the Outpatient Clinics at St Michael’s Hospital between January and November 2019. Patients underwent a physical examination and were questioned about their past medical history. Patients with documented CAD who were taking daily 81 mg ASA were asked to participate. CAD patients were defined as per American College of Cardiology, as those with stable/unstable angina, a history of NSTEMI, STEMI, or a previous coronary intervention [26]. Patients were questioned on their ASA intake and those who confirmed recent ingestion of daily 81 mg ASA for at least 14 days were recruited. Patients were excluded if they had a need for any antiplatelet therapy other than 81 mg ASA or an anticoagulant therapy, a recent diagnosis or history of thrombocytopenia, anemia or leukopenia, were clinically unwell, pregnant, breastfeeding, had a recent history (within 12 months) of NSTEMI, STEMI, or a coronary intervention, or were unable to provide written informed consent.

### 2.3. ASA Sensitivity Testing

Blood was drawn into two 2.7 mL, 3.2% sodium citrates tubes for light-transmission aggregometry (LTA) analysis and one 4 mL EDTA tube for a complete blood count. Analysis was conducted within 30 min of blood collection. LTA is the gold standard for ASA sensitivity testing with well established cutoffs in the literature, and hence was used for ASA sensitivity testing [27,28,29,30,31,32,33]. LTA was conducted as previously described [34]. Platelet-rich plasma (PRP) was prepared by centrifugation of whole blood at 300× *g* for 7 min at room temperature. A portion of the resulting PRP was further centrifuged at 1200× *g* for 10 min at room temperature to prepare platelet-poor plasma (PPP) [28,34]. Maximal platelet aggregation (light transmission) of 100% was defined as transmission through PPP, and 0% defined as light transmission through PRP. Platelet concentrations within PRP were adjusted to 2–3 × 10^6^ mL using autologous PPP. Light-transmission aggregometry was conducted on a Chrono-log aggregometer (Chrono-Log Corporation, PA, USA), at 37 °C, with stir rate set to 1000 rpm [35,36]. Platelets were activated using 0.5 mg/mL arachidonic acid (Bio/Data Corporation, Horsham, USA) reconstituted with distilled water for ASA sensitivity testing.

Baseline aggregation was analyzed for 1 min before the addition of agonist, after which the change in light transmission was analyzed for 10 min. ASA non-sensitivity was defined as ≥20% maximal platelet aggregation after the addition of arachidonic acid, as described in previous literature [27,28,29,30,31,32,33].

### 2.4. Ticagrelor Sensitivity Testing

Powdered ticagrelor (Sigma-Aldrich, Oakville, ON, Canada) was dissolved in DMSO and diluted with distilled water to obtain a ticagrelor solution. The final concentration of DMSO was <0.05% to ensure no effect of DMSO on platelet aggregation [37,38]. Patients’ PRP samples were spiked with the ticagrelor solution to obtain a final concentration of 3 μM, approximately equivalent to plasma concentrations with a loading dose of 180 mg ticagrelor [25,37,39], and was incubated for 15 min before ticagrelor sensitivity testing was conducted using LTA as previously described in Section 2.3. Platelets were activated with 5 μM ADP (Sigma-Aldrich, Oakville, ON, Canada) reconstituted with distilled water. Maximal platelet aggregation (light transmission) was calculated as described above. Previous studies have demonstrated that a ≥46% maximal platelet aggregation after activation with ADP in cardiac patients who underwent a PCI and were taking a P2Y12 receptor inhibitor was associated with higher adverse cardio-thrombotic events [40]. As per these previous studies of P2Y12 receptor inhibitors, ticagrelor non-sensitivity was defined as ≥46% maximal platelet aggregation after addition of ADP [41,42].

### 2.5. Statistical Analysis

GraphPad Prism software (GraphPad Company, San Diego, USA) v8.4.2 was used for data analysis. Demographics and clinical measurements were collected and described for the sample population. Continuous variables were tested for normality using the Shapiro–Wilk test and normality plots and expressed as mean ± standard deviation. For non-normally distributed data, the median and interquartile ranges (IQR) were calculated. Categorical variables were reported as counts or percentages. The percentages were calculated according to all patient data available. All hypothesis testing was carried out at the 5% (2-sided) significance level.

## 3. Results

### 3.1. Baseline Characteristics

A total of 38 patients with CAD who were taking daily 81 mg ASA were recruited to this study. All patients had confirmed intake of daily 81 mg ASA. The average age of patients was 69 years. Most recruited patients were males (74%) with high prevalence of hypertension (74%), hyperlipidemia (74%) and diabetes (42%) (Table 1).

Aspirin (ASA); Angiotensin-converting enzyme inhibitors (ACEi/Arb): Beta blockers (BB); Calcium channel blockers (CCB). Continuous variables are given as mean ± standard deviation and categorical variables are given as percentages. All numbers were rounded to the nearest whole number.

### 3.2. ASA Sensitivity

**Figure 1:**
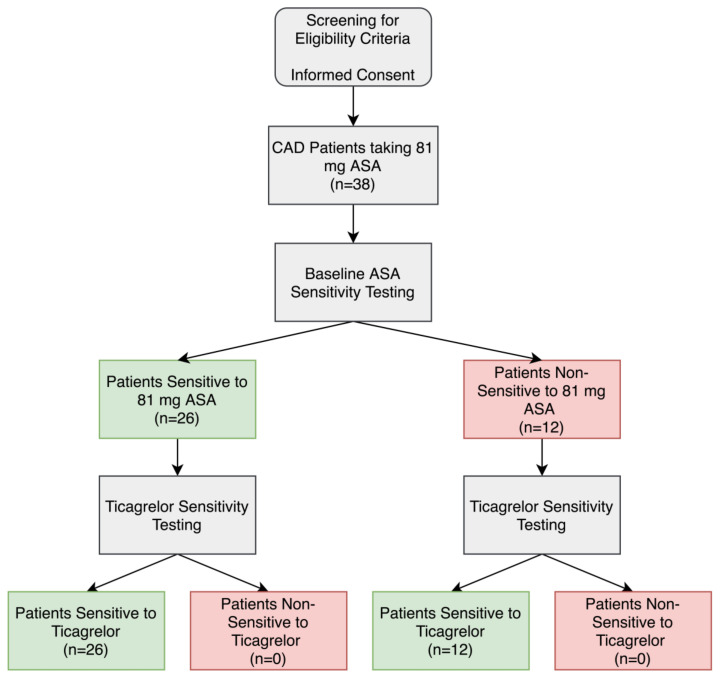
Aspirin (ASA) and ticagrelor sensitivity testing in patients taking 81 mg ASA with coronary artery disease (CAD). Patients were considered ASA non-sensitive if there was ≥20% maximal platelet aggregation following activation with arachidonic acid in light-transmission aggregometry. Patients were considered ticagrelor sensitive if there was <46% maximal platelet aggregation following activation with ADP in light-transmission aggregometry.

**Figure 2:**
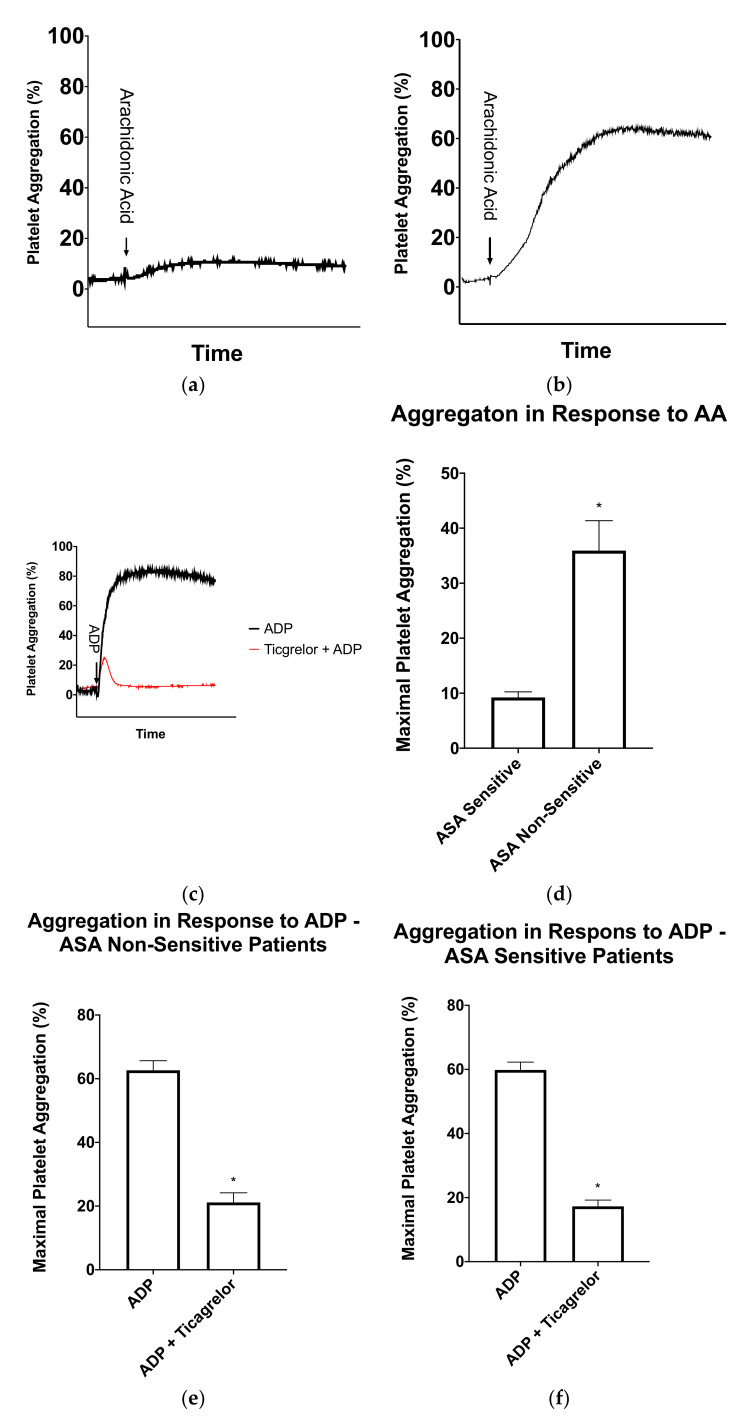
Light-transmission aggregometry results for patients taking 81 mg aspirin (ASA). (**a**) Representative light-transmission aggregometry curve of a patient sensitive to ASA and (**b**) Representative light-transmission aggregometry curve of a patient non-sensitive to ASA. (**c**) Representative light-transmission aggregometry curve of ticagrelor sensitivity testing in an ASA non-sensitive patient. The black curve represents platelet activation with ADP and the red curve represents platelet activation with ADP after a 15-min incubation with ticagrelor. (**d**) Average maximal platelet aggregation in response to arachidonic acid in ASA sensitive (*n* = 26) and ASA non-sensitive (*n* = 12) patients. (**e**) Average maximal platelet aggregation in response to ADP in the presence and absence of ticagrelor in patients non-sensitive to ASA (*n* = 12). (**f**) Average maximal platelet aggregation in response to ADP in the presence and absence of ticagrelor in patients sensitive to ASA (*figure* = 26). Error bars represent standard error of the mean. Significant difference between platelet aggregation was determined by t-test and indicated with a (*), representing *p* < 0.05 between ASA sensitive and non-sensitive patients, or between ADP platelet activation with and without ticagrelor incubation.

ASA sensitivity testing was conducted using LTA and arachidonic acid as a platelet agonist on all patients to determine the percentage of patients who were non-sensitive to their 81 mg ASA therapy. Of the 38 CAD patients, 12 patients (32%) had significantly higher maximal platelet aggregation ≥20% after arachidonic acid activation and were considered non-sensitive to 81 mg ASA (Figure 1 and Figure 2A,B,D).

### 3.3. Ticagrelor Sensitivity in ASA Non-Sensitive Patients

In order to determine if the ASA non-sensitive patients were sensitive to ticagrelor, a fresh sample of PRP was incubated for 15 min with and without 3 μM ticagrelor, a concentration similar to that seen in plasma when taking a loading dose of 180 mg ticagrelor [25,37,39]. Ticagrelor sensitivity testing was conducted with LTA using 5 μM ADP as an agonist using previously established cut-offs. In the absence of ticagrelor, patients had normal platelet aggregation in response to ADP (Figure 2C). In the presence of ticagrelor, all 12 ASA non-sensitive patients had a significant inhibition of platelet aggregation (Figure 1 and Figure 2E).

### 3.4. Ticagrelor Sensitivity in ASA Sensitive Patients

To further learn about the sensitivity of ticagrelor within our patients, we tested ticagrelor sensitivity in the 24 patients who were sensitive to ASA. As previously described, fresh PRP samples were incubated with and without 3 µM ticagrelor. Ticagrelor sensitivity testing was conducted with LTA using 5 µM as an agonist as previously described. Our data demonstrated that all 24 patients were sensitive to ticagrelor and had significantly reduced platelet aggregation in response to ADP (Figure 1 and Figure 2F).

## 4. Discussion

In our current study, we determined that 12 out of the 38 (32%) CAD patients receiving ASA as an antiplatelet were non-sensitive to their therapy and potentially at risk of further adverse cardio-thrombotic events. All 12 of these ASA non-sensitive patients were sensitive to ticagrelor ex vivo. In fact, all 38 patients who were recruited to the study were sensitive to ticgarelor.

ASA has been a staple in antiplatelet therapies provided to patients who are at risk of adverse cardio-thrombotic events [6,7,8]. It is clear, however, that a large percentage of patients are ASA non-sensitive and suffer from an adverse cardio-thrombotic event despite taking ASA for the prevention of these adverse outcomes [18]. A study conducted by Krasopouls et al. demonstrated that ASA non-sensitive patients were at a four-fold higher likelihood of suffering from another cardio-thrombotic event when compared to ASA-sensitive patients [18]. Therefore, it is important to ensure that this population of patients is receiving an effective antiplatelet therapy to prevent another adverse outcome. Ticagrelor, a “next generation” P2Y12 receptor inhibitor is an alternative antiplatelet therapy commonly prescribed to patients with CAD. It has demonstrated its efficacy as an antiplatelet medication when compared with ASA and clopidogrel [20,43]. The THEMIS trial conducted by Steg et al. in 2019 demonstrated that patients with stable CAD and diabetes benefited from taking ticagrelor in combination with ASA, compared to ASA and a placebo [23]. Ticagrelor also has significantly lower rates of non-sensitivity, as low as 0–3%, compared to ASA and clopidogrel, which may have rates as high as 65% [13,44]. As we demonstrated here in our study, 100% of our ASA non-sensitive patients were sensitive to ticagrelor. However, the THEMIS trial did note an increase in major bleeding in patients taking ticagrelor plus ASA when compared to ASA and a placebo [23]. Our study has demonstrated a method for testing for ASA non-sensitivity, and has shown that it may be beneficial to only prescribe ticagrelor to those who are non-sensitive to ASA. This could reduce the risk of minor and major bleeds, as those who are sensitive to their ASA therapy would not require being prescribed an additional antiplatelet.

Ticagrelor is a fast-acting antiplatelet, as it is an active drug, unlike its counterpart clopidogrel, which is a pro-drug requiring activation by hepatic enzymes, specifically the cytochrome P450 isoenzymes (CYPs) [45,46]. It is hypothesized that clopidogrel resistance is higher compared to ticagrelor as there are several single nucleotide polymorphisms in the CYP genes that can affect the conversion of clopidogrel into its active form [44]. The Study of Platelet Inhibition and Patient Outcomes (PLATO) compared outcomes in patients on ticagrelor or clopidogrel and noted a 16% relative risk reduction of death due to vascular causes, MI, and stroke, and a 22% relative risk reduction in overall mortality in patients taking ticagrelor; however, patients taking ticagrelor had an increase in minor, non-procedure-related bleeds when compared to patients taking clopidogrel [47]. Another study demonstrated that patients taking ticagrelor had lower rates of stroke, myocardial infarction, or death compared to patients on ASA; however, major bleeding occurred in 0.7% of patients taking ticagrelor, versus 0.4% in the ASA treated patients [48]. Therefore, if a patient is ASA non-sensitive and is being considered for ticagrelor monotherapy, the patients must be monitored for adverse bleeding.

Patients who have suffered a cardiac event and undergo cardiac surgery are often prescribed dual antiplatelet therapy, ticagrelor in combination with 81 mg ASA, for 6–12 months [49]. After this time, patients are told to discontinue one of the two antiplatelets and continue with the other. Often times physicians advise patients to stop ticagrelor and continue with 81 mg ASA, without considering whether they are ASA sensitive [49]. With our study, we have demonstrated a high percentage of ASA non-sensitivity in our sample population, and these patients may be at a higher risk of suffering from another adverse cardio-thrombotic event. With the ASA and ticagrelor sensitivity testing outlined in our study, physicians would be able to efficiently determine whether a patient is sensitive to ASA and ticagrelor, and then make a more informed decision on which drug should be discontinued. This research also demonstrates the need of further research of the possibility of using ticagrelor as a monotherapy in stable CAD, due to the high rate of ASA non-sensitivity in the population. This method of personalizing antiplatelet therapy will ensure that patients are sensitive to their therapy and receiving the antithrombotic benefits of their antiplatelet therapy.

A limitation in our pilot study is that we are testing the ex vivo response to ticagrelor in a small sample size, which may not reflect their response in vivo. Further studies are required to determine if prescribing ticagrelor to ASA non-sensitive patients is beneficial in vivo, and whether there is improved cardiovascular outcomes in these patients. It should be noted that ASA compliance was only determined by verbal confirmation from the patient, which may not necessarily reflect that the patient was truly compliant.

## 5. Conclusions

We have demonstrated using LTA, that CAD patients who are ASA non-sensitive may benefit from treatment with ticagrelor, as 100% of the ASA non-sensitive patients were sensitive to ticagrelor. Furthermore, all 38 of the patients recruited for the study were sensitive to ticagrelor ex vivo. In patients with CAD, dual antiplatelet therapy with ticagrelor and ASA has been shown to be effective; however, patients may be at a higher risk of major and minor bleeds. We have demonstrated that patients who are non-sensitive to ASA may benefit more from alternative antiplatelet therapy with ticagrelor than those sensitive to ASA. ASA sensitivity testing should be taken into consideration when deciding which antiplatelet would be most beneficial for a patient, and we have outlined an efficient method to determine if CAD patients would benefit from the prescription of ticagrelor alone or in combination with ASA.

## Figures and Tables

**Table 1:** Demographics of CAD patients taking 81 mg ASA (*n* = 38).

Mean ± SD	
Age (years)	69 ± 9
Platelet Count (103/μL)	243 ± 58
Frequency	
Sex (% Male)	28 (74)
Hypertension (%)	28 (74)
Hyperlipidemia (%)	34 (89)
Diabetes (%)	16 (42)
Renal Insufficiency (%)	3 (8)
Smokers (%)	30 (79)
Stroke (%)	0 (0)
Medication	
Statin (%)	29 (76)
ACEi/Arb (%)	18 (47)
BB (%)	17 (45)
CCB (%)	9 (24)
